# CALD Communities as “Collateral Damage” in the Criminalization of Coercive Control: An Argument for Prioritizing Civil System Reform Over Further Criminalization in Victoria

**DOI:** 10.1177/10778012231214775

**Published:** 2023-11-17

**Authors:** Balawyn Jones, Akuch Kuol Anyieth

**Affiliations:** 2080 La Trobe University, Melbourne, VIC, Australia

**Keywords:** domestic and family violence, coercive control, law reform, criminalization, marginalized communities

## Abstract

When posing the question of whether Victoria should follow other Australian states in criminalizing coercive control as a form of domestic and family violence (DFV), there are many arguments in favor of and against in the literature. However, scholars and advocates from marginalized communities, or in allyship with marginalized communities, tend to be cautious of embracing further criminalization, particularly due to the risks such an approach poses for women from culturally and linguistically diverse communities. This paper argues that women from marginalized communities are treated as “collateral damage” in the campaign to eliminate DFV via criminal law interventions.

## Introduction

Coercive control as a form of domestic and family violence (DFV) exists as a contentious topic in Victoria, which has yet to follow behind other Australian states in criminalizing it. Scholars and advocates have been cautious of embracing—or actively opposed to—further criminalization, particularly due to the risks that such an approach poses for women from culturally and linguistically diverse (CALD) communities.^1^ When acknowledging the potential harms of criminalization, namely in terms of overpolicing of marginalized communities, misidentification of the primary perpetrator, and legal systems abuse, the arguments for criminalizing coercive control are framed in a “yes, but…” fashion. Yes, these risks exist, *but* we should proceed with criminalization regardless as the potential benefits outweigh the potential risks. The question then becomes: outweighs the potential risks for whom?

In the context of Australia's colonial legal system, we argue that this reasoning positions women from marginalized communities as “collateral damage” in the campaign to eliminate DFV via criminal law interventions. Such reasoning dismisses the very real and well-documented concerns of women from marginalized communities in the pursuit of a response underpinned by carceral feminism. As part of this discussion, we analyze the civil system in Victoria which includes indirect criminalization of coercive control via breaches of family violence intervention orders (FVIOs). We argue that current issues with FVIO implementation in Victoria illustrate both a need for reform of the current system and the dangers of further criminalization, particularly for CALD communities.

### Broader Legal Context: Debates on Criminalizing Coercive Control

Coercive control is defined as a pattern of behavior that aims to dominate and control another person. Coercive control is “almost exclusively perpetrated by men against women,” in the context of both intimate partner and family relationships (ANROWS, 2021).^2^ Coercive and controlling behaviors experienced by DFV victims^3^ include “jealousy, monitoring of movements, financial abuse, social restriction, and emotional abuse or threatening behaviours” (Boxall & Morgan, 2021).

Across Australia, debates surrounding the criminalization of coercive control are currently unfolding. In Tasmania, coercive control is a stand-alone offence under ss 8 and 9 of the *Family Violence Act* 2004 (Tas). In New South Wales, the Crimes (Domestic and Personal Violence) Amendment (Coercive Control—Preethi's Law) Bill 2020 was introduced following the Joint Select Committee on Coercive Control (NSW; Parliament of New South Wales, 2020); the Bill passed on 16 November 2022 (NSW Department of Communities and Justice, 2022). In Queensland, the Domestic and Family Violence Protection (Combating Coercive Control) and Other Legislation Amendment Bill 2022 was brought before parliament for debate following the Women's Safety and Justice Taskforce (Qld; Queensland Women's Safety and Justice Taskforce, 2021). In South Australia, the Criminal Law Consolidation (Abusive Behaviour) Amendment Bill 2023 was introduced following release of an exposure Bill (SA; YourSAy, 2021). At the time of writing, in Western Australia, a law reform process led by the Commissioner for Victims of Crime (WA) was currently underway (Western Australia Commissioner for Victims of Crime, 2023).

In Victoria, the 2016 Royal Commission into Family Violence (Vic) did not recommend “the introduction of a stand-alone offence of coercive control” (Fitz-Gibbon et al., 2020). However, “in recent years momentum has begun to push for a reconsideration” of this position (Monash Gender and Family Violence Prevention Centre, 2020). For example, in November 2021, the Victorian Legislative Council passed a motion which “recognised the ‘prevalence of coercive control in family violence offending’, and that called on the government to look into ways to ‘enhance the understanding of coercive and controlling behaviour in our community and the justice system’” (Otter et al., 2021). It is against this background that our article makes its intervention. Given that the tide in the Australian legal and political context is turning toward favoring a criminalization approach, we argue that Victoria's initial position against further criminalization of coercive control should be maintained. Further, we argue that Victoria's existing system of indirect criminalization of coercive control as part of civil law FVIOs should be reformed instead of pursuing further criminalization in the form of a stand-alone offence.

### Broader Theoretical Context: Carceral Feminist Approaches to DFV

Contemporary debates relating to the criminalization of coercive control are in many ways not new but build upon decades of feminist debate between carceral versus abolitionist approaches to combatting violence against women, including the notable contributions and critiques of Black feminists and First Nations feminists. To provide context, the label *carceral feminism* was devised by sociologist Elizabeth Bernstein, who critiqued overreliance on police and prisons (which reinforce State violence) as the “solution” to DFV (Bernstein, 2007). Carceral feminism is based on the assumption that violence against women is caused by isolated individuals and, therefore, individual punishment or exclusion is the only and best option available, as well as assuming that the State's legal systems are mostly fair (Bernstein, 2007; Gruber, 2023).

Reform initiatives underpinned by carceral feminism often aim to create stronger laws, policies, and procedures, accompanied by increased policing, prosecution, and imprisonment. Critiques of carceral feminism include abolitionist feminist arguments against the application of solely or predominately punitive approaches to combat violence against women, including the consequences that such policies have had in terms of mass incarceration (Kim, 2018). Julia Quilter (2020) has described this overreliance on criminalization as a “gap-filling” paradigm. Relying on Quilter, Jane Wangmann further explains that the risk of viewing a new criminal offence as “*the* measure that will fill *the* gap” is that it “‘may impliedly endorse the idea … that physical family violence is currently well policed and adequately addressed by the criminal law” (Wangmann, 2022, citing Quilter, 2020). Critiques of carceral feminism tend to point to systematic failures in our criminal law systems, particularly in law enforcement, which would be overlooked or entrenched by the creation of a new criminal offence as opposed to reforms aiming to address underlying issues within our legal systems. In addition to these critiques, carceral feminist approaches are often associated with a white, middle-class lens that inherently excludes the perspectives of marginalized communities, including a lack of engagement with CALD groups and their lived experience of the law (which will be discussed further below).

The debates relating to carceral versus abolitionist feminism also build on literature related to the role of the State and (criminal) law more broadly. For example, Black feminist scholars such Angela Davis and bell hooks have argued that advocates for carceral solutions to DFV tend to rely on state-based approaches for addressing violence against women without critically engaging with the structural violence of social inequality, racism, capitalism, and imperialism (Smith, 2008, citing Davis, 1981 and hooks, 1981).^4^ Such approaches fail to address the role of the State in initiating and perpetuating violence against women, particularly poor women, women of color, and women from migrant and refugee communities. These critiques are especially relevant in the Australian context where First Nations scholars and advocates have highlighted the continuing legacy of colonial violence through law, including in the DFV space (Watego et al., 2021).^5^ More broadly, Aida Hurtado identifies how white privilege in the feminist space has allowed white women to become the most visible (and prioritized) voices in setting the feminist agenda, including in terms of laws that respond to DFV—that is, the paradox of white carceral feminism, where white women remain racially privileged despite being gender subordinated (Hurtado, 1989; see also Masson, 2020).

In the context of coercive control debates, First Nations scholars and organizations such as the Victorian Aboriginal Legal Service (VALS) have highlighted the risks of a criminalization approach for marginalized communities, especially Aboriginal and Torres Strait Islander people. VALS outlines how a new offence would have “disproportionate impacts on Aboriginal people in Victoria,” including the misidentification of DFV victims as primary perpetrators and overpolicing of Aboriginal people due to “systemic racism” in policing (Victorian Aboriginal Legal Service, 2022). The VALS position paper concludes that:

… deep problems with [the Victorian] police's approach to Aboriginal people in Victoria persist, and these continue to affect the police response to family violence. In this context, the likelihood that a new criminal offence would deliver any protection or benefit to Aboriginal women is extremely low. (Victorian Aboriginal Legal Service, 2022)

Criminalization of coercive control is likely to result in either a lack of protection for DFV victims who belong to marginalized communities or cause further harm (One Woman Project, 2021). The assertion that further criminalization is likely to cause further harm to marginalized communities is based on decades of evidence which documents the disproportionate negative impacts of criminal laws on marginalized communities including Aboriginal and Torres Strait Islander and CALD victims of DFV (Mantle & Hermans, 2020; Nancarrow et al., 2020; Victorian Aboriginal Legal Service, 2022; Watego et al., 2021).

Based on the legacy of abolitionist feminist literature discussed in this section, in the next section, we argue that further criminalization of coercive control and the accompanying increases in policing and State power will, at best, not protect victims from marginalized communities and at worst, actively cause harm to CALD communities. In doing so, we look at the CALD-specific barriers to access to justice in the DFV space and how these barriers relate to the criminalization of coercive control.

### More Than Collateral Damage—CALD Perspectives on the Criminalization of Coercive Control

Women who speak a language other than English at home are among the three groups of women who are “significantly overrepresented” in terms of experiencing coercive control—the other two being Aboriginal and Torres Strait Islander women and women who have disabilities (Boxall & Morgan, 2021; Otter et al., 2021). While the exact “prevalence of family violence within CALD communities is not readily quantifiable due to under-reporting and barriers to disclosure,” it is known that CALD-specific circumstances “significantly compound barriers to access the right services and support in the event of family violence occurring, and may contribute to increased risk of violence” (Victorian Multicultural Commission, 2015).^6^ As explained below, these circumstances include: language barriers, cultural barriers, migration pathways, social isolation, racism, discrimination, and the double bind.

To provide context, according to 2021 Australian census data, 27.6% of Australians were born overseas, 48.2% of Australians have at least one parent who was born overseas, and 24.8% of Australians speak a language other than English at home (ABS, 2021a, 2021b). Multiple intersecting identities exist under the label of “Culturally and Linguistically Diverse Communities.” As explained in Muslim Women Australia's submission to the NSW Joint Select Committee on Coercive Control:

Intersectional identity markers impacting the way CALD women experience the world include, but are not limited to, ‘first language spoken, visible minority status, migration status, religious affiliation… (Muslim Women Australia, 2021)

CALD and faith-based communities are diverse and not all members of these communities will be affected to the same degree by these factors … For example, CALD women have vastly different experiences depending on whether they are a first or second-generation migrant, the life stage they were at when migration took place, whether they are a recent migrant/refugee or depending on their visa pathway. CALD women are not only from newly arrived migrant communities but extend across the spectrum of settlement including established communities. (Muslim Women Australia, 2021)

In acknowledging this diversity, it goes without saying that advocates within the CALD community and other marginalized communities differ in terms of their approach toward the criminalization of coercive control. For example, some organizations (such as InTouch Multicultural Centre Against Family Violence) have expressed in-principle support for the criminalization of coercive control. However, such support is conditional on “whole of system change,” without which the criminalization of coercive control is likely to have “far-reaching unintended consequences, particularly for vulnerable individuals and marginalised communities” (InTouch, 2021. See also Buxton-Namisnyk et al., 2022). In contrast, other communities have explicitly advocated against further criminalization. As explained above, the strongest abolitionist advocacy comes from First Nations scholars and organizations.

Arguments for criminalizing coercive control tend to center around ensuring that the law accurately reflects the lived experience of DFV victims, encompassing both physical and nonphysical forms of violence over time, as well as advancing the law to align with community expectations and needs (Australian Women Against Violence Alliance, 2021; Gleeson, 2019).^7^ Some advocates argue that criminalization will assist law enforcement to prosecute cases involving nonphysical violence (solely or in combination with physical violence) and overcome current gaps in the law (Barlow et al., 2020; Burman & Brooks-Hay, 2018). Another aim of criminalization is to “send a message” that nonphysical forms of DFV are just as harmful as physical abuse and that such behavior will not be tolerated in the Victorian community (Walklate & Fitz-Gibbon, 2021).^8^

Based on the abolitionist critiques of prioritizing some victim and advocate voices over others and critiques of a gap-filling approach to dealing with complex social phenomena through an overreliance on criminal law, we argue that the above justifications for criminalization are inadequate given the tangible harms that criminalization poses to marginalized communities and the well-documented issues with implementation of the existing Victorian approach; that is, indirect criminalization of DFV (discussed below).

### Language Barriers—the Risk of Misidentification of the Primary Perpetrator

The most obvious barrier faced by many CALD victims is that of language. While not all people from CALD communities experience language barriers to the same extent (if at all), where this is a relevant factor, it can greatly impact DFV victims’ access to justice. Language barriers can impact access to justice when interpreters or translators are not made available by service providers or where inappropriate interpreters or translators are used, resulting in “lost information, and ultimately loss of confidence in the justice system” (Victorian Multicultural Commission, 2015).

As explained by Muslim Women Australia, in the context of criminalizing coercive control:

… a potential disadvantage of criminalising coercive control is the risk of systems abuse due to misidentification of the primary aggressor. For example, in CALD communities if the perpetrator has better English and speaks to police, or the victim's account of events is not facilitated by an interpreter, then the victim may be misidentified as the perpetrator. Police routinely do not provide interpreters and multicultural communities have tried to engage with police many times on these issues. (Muslim Women Australia, 2021)

It is well-documented, in the context of DFV service provision (including policing), that the current availability of “professional and independent interpreting and translating services is inadequate” (Victorian Royal Commission into Family Violence, 2016).

### Social Isolation and Migration Status—the Risk of Legal Systems Abuse

The migration pathway or type of visa that a DFV victim holds can significantly impact access to justice, particularly for women who have newly migrated or who are not permanent residents. This is because DFV victims may be “dependent on the perpetrator for residential or citizenship status” (Victorian Multicultural Commission, 2015), and may be “fearful of losing the right to reside in Australia” or “fearful of losing custody of their children” if they are deported (InTouch, 2018).^9^ Perpetrators may actively use victims’ migration status as a part of coercive and controlling behavior, for example, by confiscating their passports or making threats related to deportation (InTouch, 2018). Further, due to either social isolation or the nature of their visa, women who are new migrants may have limited access to (or knowledge of) Australian legal or social support systems (Muslim Women Australia, 2021; National Domestic and Family Violence Bench Book, 2022).^10^

Depending on the individual circumstances of the victim, their migration pathway may also relate to a “lack of financial stability or independence” (Muslim Women Australia, 2021). It should be noted that the CALD-specific barriers to access to justice exist in addition to general barriers to access to justice faced by DFV victims, such as socioeconomic barriers and regional or rural isolation. General barriers to access to justice may also be compounded when intersecting with CALD-specific barriers. Due to social isolation and a lack of access to relevant supports, CALD victims who are newly migrated or who are not permanent residents are “at greater risk of coercion and control by sponsoring spouses and other family members” (Victorian Royal Commission into Family Violence, 2016). In terms of coercive control, women from CALD backgrounds may face legal systems abuse at the intersection of DFV and immigration law.

### Racism and Discrimination—The Risk of Overpolicing

It is well-documented that “many CALD women face racism and discrimination when seeking help through mainstream DFV service providers. This includes service providers’ assumptions about violence being a part of CALD community cultures and negative stereotypes about CALD or Muslim communities” (Muslim Women Australia, 2021, citing ‌InTouch, 2010; ANROWS, 2021; University of Queensland Pro Bono Centre, 2020). One of the barriers unique to women from marginalized communities when speaking out about DFV or advocating on issues relating to the criminalization of coercive control is the double bind. The double bind is “the meeting point between Islamophobia/imperialism and gender injustice, in which women find themselves subject to criticism both within and beyond their communities in the fight for gender justice” (Muslim Women Australia, 2021, citing Hussein, 2010). Many victims of DFV, as well as advocates operating in this space, are wary of the “consequences of speaking out in relation to abuse” (Victorian Multicultural Commission, 2015). These consequences exist as both potential criticism or backlash from within their communities and the fact that speaking out in relation to abuse within their communities may lead to further stigmatization of or discrimination against their communities.^11^

Against the background of overpolicing and racism in policing (outlined in the section above), “fear and distrust of the police, justice system and support services often dissuade CALD women from reporting family violence” (Victorian Multicultural Commission, 2015). Further, one of the biggest risks posed by the criminalization of coercive control is that such an approach will result in further overpolicing of marginalized communities (Domestic Violence Resource Centre Victoria, 2021; InTouch, 2021). As with First Nations women, “women from migrant and refugee communities are more likely to be misidentified as the predominant aggressor than other women in the community” (InTouch, 2021, citing No To Violence, 2019).^12^ In 2021, the Victorian Implementation Monitor (following on from the Royal Commission into Family Violence) found that “misidentification continues to occur, and rectification is extremely challenging” (Wangmann, 2022, citing Shuard, 2021).

### Cultural Barriers—The Need for Community-Based Solutions Rather Than Criminalization

Although many forms of DFV that occur within CALD communities are similar to, or the same as, those that occur within the broader Anglo-Australian community, some forms of DFV are specific to or more prevalent among CALD communities (InTouch, 2018). For instance, violence associated with dowry abuse, forced marriage, honor killings, and female genital mutilation is often culturally specific (InTouch, 2018). Further, violence perpetrated by extended family members may be more common in CALD communities, as women often move in with their husband's extended family members (InTouch, 2018). Women from CALD communities may also face unique barriers to economic independence related to cultural beliefs or practices that make women financially dependent on their husbands and their husband's families (Cocodia & Dedeigbo, 2016; InTouch, 2018). In addition, in some CALD communities, a higher cultural value is placed on protecting the family and family reputation above individual rights (Kalapac, 2016). Victims from such backgrounds may find it challenging to report violence or approach DFV services as they fear family honor could be jeopardized, which can result in family and community rejection or stigmatization (Kalapac, 2016). This causes hesitation in reporting violence or pursuing other avenues such as separation or divorce (InTouch, 2018; Pittaway et al., 2011).^13^

However, cultural beliefs and practices are not uniform across different CALD communities. Given the diversity of CALD communities, the extent to which the concept of coercive control can effectively capture CALD women's lived experience of DFV, and the different forms and dynamics of violence, is open to debate. This raises the problem of interpreting what coercive control means for individuals in CALD communities and the potential for cultural misinterpretations and barriers to seeking help (Bishop, 2016; Walklate et al., 2018).

Women from CALD communities often do not report DFV due to their own cultural perceptions and understandings as to what constitutes DFV in terms of physical violence, let alone what constitutes coercive control or nonphysical violence (Australian Women Against Violence Alliance, 2021; Barlow et al., 2020). In many CALD communities, understandings of DFV exclude emotional, psychological, and even sexual abuse (InTouch, 2018). More resources need to be invested in DFV education and awareness raising in CALD communities to challenge and change community interpretations of DFV before pursuing criminalization.

Perpetrator programs such as Men's Behavior Change (MBC) programs are also inadequate for many CALD perpetrators and do little to effect change in perpetrator behaviors as they are generally based on Anglo-Saxon understandings and practices. Existing court-ordered programs in combination with FVIOs, such as alcohol and drug counseling and MBC programs, are often based on the Duluth model, which heavily reflects Western views regarding DFV (Crichton-Hill, 2001; Department of Families, Fairness and Housing, 2019). Studies have shown that perpetrators in general—from either Western or non-Western backgrounds—rarely seek help to address their violent behavior (Corbally, 2015; Machado et al., 2017; Mugford, 1989; Taylor et al., 2022; Tsui et al., 2010). This resistance may also be influenced by cultural beliefs and practices for CALD men (Victorian Foundation for Survivors of Torture, 2015; Victorian Government, 2022). Barriers to engagement with MBC programs are further exacerbated by language barriers and social inequalities for CALD perpetrators, which limits their access to support and results in the continuation or escalation of violence in their life (Multicultural Centre for Women's Health, 2015).

While it is recognized that language, cultural, and systemic barriers are not an excuse for violence, there is a need to improve current DFV perpetrator intervention measures for those from different cultural backgrounds (Australian Institute of Health and Welfare, 2018). Evidence shows that, due to cultural, socioeconomic, and language barriers and a lack of understanding about available DFV services (for both victims and perpetrators), the current education and prevention programs are often ineffective in CALD contexts (Domestic Violence Resource Centre Victoria, 2021; Fitz-Gibbon et al., 2020; Victorian Aboriginal Legal Service, 2022; Women's Legal Services Victoria, 2020). These fundamental failures in existing service provision require attention. Further investment in community-based solutions could contribute to a noncarceral, nonviolent, and potentially preventative approach to coercive control and DFV within CALD communities. In other words, the current literature points to the importance of justice reinvestment measures—rather than a need for further criminalization (Otter et al., 2021).^14^

In light of the seriousness of the CALD-specific barriers discussed above—in particular, the exacerbated risks of legal systems abuse, misidentification of the primary perpetrator, and overpolicing, as well as evidence pointing toward a need for community-based solutions rather than criminalization—it can be questioned whether the majority of advocacy in favor of criminalizing coercive control can be said to have seriously engaged with CALD perspectives. It is for these reasons, as well as the reasons below, that we categorize much of the contemporary debate relating to the criminalization of coercive control as treating CALD women as acceptable “collateral damage” in the pursuit of a carceral feminist response to DFV.

## Challenging the Presumption of Criminal Law's Utility—An Analysis of FVIOs in Victoria

Building on the analysis above, in this section we highlight the failures and successes of indirect criminalization of coercive control which currently exist within the Victorian civil system. By highlighting the failures of the current system, we intend to present further evidence in terms of why a stand-alone criminal offence would not “solve” the problem of coercive control as well as to highlight opportunities for reform of the current system. Overall, we argue that reform of the current civil law system is a preferable avenue compared to pursuing further criminalization in the Victorian context.

### Victoria's Current Approach

Unlike in other States and territories, Victoria's legal system already recognizes coercive control, albeit within the civil law system rather than the criminal law system.^15^ In Victoria:

… coercive, controlling and dominating behaviour is enshrined in the definition of family violence within the *Family Violence Protection Act (Vic)* 2008 (the FVPA) and its Preamble, which sets out the principles underpinning the Act including recognising family violence as ‘patterns of abuse’ that occur over time. … The principles set out in the Preamble, the purpose, and the definition of family violence in the FVPA guide legal, policy and practice frameworks in Victoria and means that if a victim-survivor is experiencing coercive and controlling behaviours, there is recourse through the civil jurisdiction as a victim-survivor or Victoria Police can apply for a Family Violence Intervention Order (FVIO). (Domestic Violence Resource Centre Victoria, 2021)

Once granted, a breach of the conditions of an FVIO constitutes a criminal offence (Domestic Violence Resource Centre Victoria, 2021; Women's Legal Service Victoria, 2020).^16^

We argue that the recognition, and indirect criminalization, of coercive control under Victoria's FVIO system means that there is no need for further criminalization,^17^ both in terms of there being no “gap” to be filled as well as limiting the weight of arguments that advocate for a new criminal offence based on its “normative value.” Given the risks of further criminalization and the strength of community-based solutions as alternatives to criminalization (flagged in the section above), this section will explain how reform of indirect criminalization of coercive control via FVIOs should be prioritized in lieu of creating a stand-alone offence in the Victorian context.

### Critiques of the Current System From a CALD Perspective and Implications for Further Criminalization

With more than 12,000 FVIOs applied for in Victoria each year (Victoria Police, 2022), such intervention orders are one of the most commonly used forms of intervention by law enforcement in DFV cases. Although they are the most used form of intervention, FVIOs are often breached. According to the Sentencing Advisory Council, there were 316,668 FVIO breaches between 2011 and 2020 (Sentencing Advisory Council, 2022). Due to the number of breaches, there is no denying that, in terms of policing, FVIOs often fail to protect victims from DFV and change needs to occur.

#### Language and Cultural Barriers

The barriers to access to justice for CALD communities (outlined above) equally apply to the issuing and enforcing of FVIOs. For CALD communities, language barriers and a limited understanding of Australian legal systems have been identified as reasons why both perpetrators and victims of DFV breach FVIO orders (Centre for Innovative Justice, 2021). In addition to a general lack of legal knowledge and English language barriers, breaches can also be related to a lack of understanding or misinterpretation of the specific conditions in the order and a lack of proper legal counsel or inadequate time with a legal representative to adequately explain the conditions of the FVIOs to all parties (Centre for Innovative Justice, 2021).

For people from CALD communities where English is a second language, there should be more comprehensive processes in place to ensure that both perpetrators and victims receive adequate support to understand the conditions of the FVIO. However, in many instances, this is not currently the case. When FVIOs are served in the absence of proper legal counsel or interpretation/translation services, CALD parties with intersecting social and legal issues are often left open to the likelihood of the orders being breached, sometimes inadvertently. For instance, it is usual for CALD family members (including broader kinship networks) to live within close proximity. However, this dynamic can lead to a breach of an FVIO in instances where a perpetrator visits another family member or relative while the victim is present. Another example is where cultural dynamics related to places of worship, such as churches and mosques, mean that both parties attend a communal location. Further reasons for breaches of FVIOs include broader socioeconomic barriers which may affect (but are not unique to) CALD communities, including “homelessness, poverty, drug and alcohol issues, [and] mental health issues” (Centre for Innovative Justice, 2021).

#### Overpolicing and Discrimination

For existing interventions and preventative measures to achieve their intended purposes, there must be considerable improvements and changes to the working relationship between police and CALD communities. As mentioned earlier, systemic racism has been documented in policing, including in the context of DFV, particularly against First Nations people (Victorian Aboriginal Legal Service, 2022). VALS has identified a lack of cultural competency in the current Victorian approach to coercive control, including the inclusion of “inappropriate conditions” in FVIOs which set perpetrators and victims (who have been misidentified as the primary perpetrator) up for failure, further drawing them “into contact with the criminal legal system” (Victorian Aboriginal Legal Service, 2022, citing Douglas & Fitzgerald‌, 2018). In addition, Ellen Reeves argues that misidentification of the primary perpetrator under the current civil system disproportionately impacts “marginalised populations including Aboriginal and Torres Strait Islander (ATSI) women, women living with disabilities and culturally and linguistically diverse (CALD) women” (Reeves, 2021).

The literature in this area highlights numerous unresolved barriers to the successful implementation of indirect criminalization via the Victorian civil law system. We argue that current barriers to the implementation of FVIOs should be addressed instead of pursuing the creation of a new criminal offence. Further, the Royal Commission into Family Violence in Victoria expressly noted that “education, training and embedding best practice and family violence specialization in the courts is likely to be more effective than simply creating new offences or changing sentencing laws” (Domestic Violence Resource Centre Victoria, 2021, citing Victorian Royal Commission into Family Violence, 2016). This finding emphasizes the need for investment in improved education and training, rather than a need for more law. We also argue that outside of the formal legal system (i.e., policing and courts), there is an acute need for investment in community-based solutions consistent with justice reinvestment principles.

## Conclusion

In this article, we have sought to demonstrate that arguments for the criminalization of coercive control tend to marginalize CALD perspectives and downplay or ignore evidence of the risks of criminalization for CALD communities. In the second section, the risks of further criminalization from a CALD perspective were documented, building on broader abolitionist critiques of carceral approaches in the DFV context. The third section sets out the current issues with indirect criminalization via FVIO implementation in Victoria, illustrating both a need for reform of the current system and the risks of criminalization in general.

We argued that Victoria should pursue reform of the current FVIO civil system instead of pursuing stand-alone criminalization of coercive control. The introduction of a new law criminalizing coercive control would result in further harm to men, women and children from CALD communities, rather than resulting in better protection of DFV victims. Further, as social, economic, language, and cultural barriers currently inhibit the goals of achieving women and children's safety and perpetrator accountability, we argue that resources and efforts should be dedicated toward holistic and culturally competent, community-based solutions.

## Notes

1. “Culturally and Linguistically Diverse” (CALD) is a term used to refer to marginalized ethnic and racial groups within Australian legal and policy literature, alongside the terms “Culturally and Racially Marginalized” (CARM) and “ethnic minorities.” The term “CALD” is generally understood as encompassing non-Anglo-Saxon ethnic and racial minorities other than First Nations communities. First Nations communities are generally distinguished from CALD communities, with CALD referring to migrant communities rather than indigenous communities. While the authors acknowledge that the term CALD is problematic in and of itself, it is currently the prevailing terminology in the field.

2. Note that while DFV may impact all people, including trans-men and -women, intersex and nonbinary people, this article focuses on DFV between cis-men and -women in heterosexual relationships, the dynamics of DFV in LGBTQI+ relationships are outside the scope of this article. The data relating to DFV in both CALD communities and LGBTQI+ contexts are limited; due to this lack of data very little literature speaks to the intersection of these two categories in the Australian context. For contemporary literature on coercive control in LGBTQI+ relationships see, for example, Reeves et al. (2023).

3. As explained by Jones et al. (2022): The word “victim” is used in this article as opposed to “survivor” to acknowledge the harms committed against, and experienced by, women in DFV contexts. Importantly, the label “victim” should not be equated with passivity or weakness in a false binary, with “agency,” on the other hand, associated with strength or courage. To do so fails to recognize that agency must be viewed within the frame of victimization and vice versa. For a theoretical discussion of the victim/agent dichotomy in the Acehnese context, see Jones, 2020, particularly 173–175.

4. See also Davis (2022).

5. See also: Lean et al. (2021); Institute for Collaborative Race Research & Sisters Inside (2021); Robinson (2022).

6. See also Victorian Royal Commission into Family Violence (2016): “People from culturally and linguistically diverse communities are more likely than people of Anglo-Australian background to face barriers to obtaining help for family violence.”

7. See also forthcoming research by Fitz-Gibbon, Walklate, Meyer, and Reeves on the complexity of victim-survivors’ views on the need for, benefits, risks, and impacts of criminalization.

8. See also Otter et al. (2021).

9. See also Muslim Women Australia (2021) where victims raised concerns regarding their immigration status and fear of losing custody of their children if they reported DFV.

10. Muslim Women Australia (2021) raises issues including a lack of familial and social support networks and lack of knowledge about the Australian legal system; the National Domestic and Family Violence Bench Book (2022) further mentions a lack of knowledge of Australian law and available services for CALD communities as barriers to access to justice.

11. See Abu-Lughod (2015).

12. Also see Domestic Violence Resource Centre Victoria (2021).

13. See also Wright (2023), who highlights how DFV victims in CALD community tend to report within their own communities through informal networks as a “first port of call.”

14. Otter et al. (2021), citing Barclay (2020): “[C]arceral responses to domestic violence should be replaced with economic, public health, community-based and human rights solutions.”

15. Women's Legal Service Victoria (2020): The law in Victoria recognizes that coercive control is family violence. Coercive control is a central feature of Victoria's civil law *Family Violence Protection Act* (2008). In its definition of family violence, the Act recognizes that family violence includes coercive behavior that controls or dominates a family member—and includes emotional, psychological, and economic abuse. Family violence is defined in Victoria's *Family Violence Protection Act* (2008) as: (a) behavior by a person toward a family member of that person if that behavior (i) is physically or sexually abusive; or (ii) is emotionally or psychologically abusive; or (iii) is economically abusive; or (iv) is threatening; or (v) is coercive; or (vi) in any other way controls or dominates the family member and causes that family member to feel fear for the safety or well-being of that family member or another person; or (b) behavior by a person that causes a child to hear or witness, or otherwise be exposed to the effects of, behavior referred to in paragraph (a).

16. Domestic Violence Resource Centre Victoria (2021): “If any of the ‘conditions’ on the FVIO are subsequently breached by the perpetrator, it is considered a criminal offence, and this acts as a pathway to the criminal justice system.” See also Women's Legal Service Victoria (2020): “The *Family Violence Protection Act* (2008) ensures a person is held criminally accountable where a Family Violence Intervention Order is breached.”

17. Victorian Aboriginal Legal Service (2022): “the arguments against criminalisation are even stronger in the context of Victoria's existing legislative, policy and operational framework for responding to family violence. The potential benefits of adding a criminal offence on top of this existing framework are minimal.” In other words, we argue that the risks outweigh the benefits in the Victorian context.

## References

[bibr1-10778012231214775] Abu-Lughod L. (2015). Do Muslim women need saving? Harvard University Press.

[bibr2-10778012231214775] ANROWS. (2021). *Defining and responding to coercive control: Policy brief*. https://www.anrows.org.au/publication/defining-and-responding-to-coercive-control/.

[bibr3-10778012231214775] Australian Bureau of Statistics. (2021a). *Census data 2021*. https://www.abs.gov.au/census/find-census-data/quickstats/2021/AUS.

[bibr4-10778012231214775] Australian Bureau of Statistics. (2021b). *Media releases*. https://www.abs.gov.au/media-centre/media-releases/2021-census-nearly-half-australians-have-parent-born-overseas.

[bibr5-10778012231214775] Australian Institute of Health and Welfare. (2018). *Intimate partner violence in Australian refugee communities: Scoping review of issues and service responses*. https://apo.org.au/sites/default/files/resource-files/2018–12/apo-nid211166.pdf.

[bibr6-10778012231214775] Australian Women Against Violence Alliance. (2021). *Criminalisation of coercive control: Issues paper*. https://awava.org.au/2021/01/28/research-and-reports/criminalisation-of-coercive-control-issues-paper.

[bibr7-10778012231214775] Barclay P. (2020). Interview with Leigh Goodmark. Big ideas. ABC Radio National.

[bibr8-10778012231214775] Barlow C. Johnson J. Walklate S. Humphreys L. (2020). Putting coercive control into practice: Problems and possibilities. The British Journal of Criminology, 60(1), 160–179. 10.1093/bjc/azz041.

[bibr9-10778012231214775] Bernstein E. (2007). The sexual politics of the “new abolitionism”. Differences, 18(3), 128–151. 10.1215/10407391-2007-013.

[bibr10-10778012231214775] Bishop C. (2016). Why it’s so hard to prosecute cases of coercive or controlling behaviour. *The Conversation*. https://theconversation.com/why-its-so-hard-to-prosecute-cases-of-coercive-or-controlling-behaviour-66108.

[bibr11-10778012231214775] Boxall H. Morgan A. (2021). Experiences of coercive control among Australian women. Australian Institute of Criminology. https://www.aic.gov.au/publications/sb/sb30 .

[bibr12-10778012231214775] Burman M. Brooks-Hay O. (2018). Aligning policy and law? The creation of a domestic abuse offence incorporating coercive control. Criminology and Criminal Justice, 18(1), 67–83. 10.1177/1748895817752223.

[bibr13-10778012231214775] Buxton-Namisnyk E. Gibson A. MacGillivray P. (2022). Unintended, but not unanticipated: Coercive control laws will disadvantage First Nations women. *The Conversation*. https://theconversation.com/unintended-but-not-unanticipated-coercive-control-laws-will-disadvantage-first-nations-women-188285.

[bibr14-10778012231214775] Centre for Innovative Justice. (2021). *More than just a piece of paper. Getting protection orders made in a safe and supported way: Responding to Recommendation 77 of the Royal Commission into Family Violence*. https://cij.org.au/cms/wp-content/uploads/2018/08/more-than-just-a-piece-of-paper-summary-report-2021.pdf.

[bibr15-10778012231214775] Cocodia E. Dedeigbo O. (2016). Domestic violence in Australia’s CALD communities: Association between demographics of frontline workers and selected therapeutic approaches. Asia Pacific Institute of Advanced Research. http://apiar.org.au/?conference-paper=domestic-violence-in-australias-cald-communities-association-between-demographics-of-frontline-workers-and-selected-therapeutic-approaches .

[bibr16-10778012231214775] Corbally M. (2015). Accounting for intimate partner violence: A biographical analysis of narrative strategies used by men experiencing IPV from their female partners. Journal of Interpersonal Violence, 30(17), 3112–3132. 10.1177/0886260514554429. 25392374

[bibr17-10778012231214775] Crichton-Hill Y. (2001). Challenging ethnocentric explanations of domestic violence. Trauma, Violence & Abuse, 2(3), 203–214. 10.1177/1524838001002003001.

[bibr18-10778012231214775] Davis A. (1981). Women, race, and class. Random House.

[bibr19-10778012231214775] Davis A. (2022). Abolitionism. Feminism. Now. Penguin Books.

[bibr20-10778012231214775] Department of Families, Fairness and Housing. (2019). Evaluation of new community-based perpetrator interventions and case management trials. Victorian Government. https://www.vic.gov.au/evaluation-perpetrator-interventions-final-report .

[bibr21-10778012231214775] Domestic Violence Resource Centre Victoria. (2021). *Responding to coercive control in Victoria – Broadening the conversation beyond criminalisation*. https://safeandequal.org.au/wp-content/uploads/PAP_202105_Responding-to-Coercive-Control_FINAL.pdf.

[bibr22-10778012231214775] Douglas H. Fitzgerald R. (2018). The domestic violence protection order system as entry to the criminal justice system for Aboriginal and Torres Strait Islander people. International Journal for Crime, Justice and Social Democracy, 7(3), 41–57. 10.5204/ijcjsd.v7i3.499.

[bibr23-10778012231214775] Fitz-Gibbon K. Walklate S. Meyer S. (2020). Australia is not ready to criminalise coercive control – Here is why. *The Conversation*. https://theconversation.com/australia-is-not-ready-to-criminalise-coercive-control-heres-why-146929. 10.1177/10778012231220370 PMC11792385 38105507

[bibr24-10778012231214775] Fitz-Gibbon K. Walklate S. Meyer S. Reeves E. (forthcoming). The criminalisation of coercive control: A national study of victim-survivors’ views on the need for, benefits, risks and impacts of criminalisation. Monash University. https://www.monash.edu/arts/gender-and-family-violence/research-and-projects/changing-legal-responses-to-family-violence .

[bibr25-10778012231214775] Gleeson H. . (2019). Coercive control: The ‘worse part’ of domestic abuse is not a crime in Australia. But should it be? *ABC*. https://www.abc.net.au/news/2019-11-19/coercive-control-domestic-abuse-australia-criminalise/11703442.

[bibr26-10778012231214775] Gruber A. (2023). The critique of carceral feminism. Yale Journal of Law and Feminism, 32(2), 55–64. http://hdl.handle.net/20.500.13051/18248.

[bibr27-10778012231214775] hooks b. (1981). Ain't I a woman?: Black women and feminism. South End Press.

[bibr28-10778012231214775] Hurtado A. (1989). Relating to privilege: Seduction and rejection in the subordination of white women and women of color. Signs, 14(4), 833–855. http://www.jstor.org/stable/3174686. https://doi.org/10.1086/494546.

[bibr29-10778012231214775] Hussein S. (2010). Double bind and double responsibility: Speech and silence among Australian Muslim women. In Akbarzadeh S. (Ed.), Challenging identities: Muslim women in Australia. Melbourne University Press.

[bibr30-10778012231214775] Institute for Collaborative Race Research & Sisters Inside. (2021). *The state as abuser: Coercive control in the colony. Joint submission from Sisters Inside and the Institute for Collaborative Race Research on Discussion Paper 1 of the Women’s Safety and Justice Taskforce*. https://www.sistersinside.com.au/the-state-as-abuser-coercive-control-in-the-colony/.

[bibr31-10778012231214775] InTouch Multicultural Centre Against Family Violence. (2010). *Barriers to the justice system faced by CALD women experiencing family violence*. InTouch Multicultural Centre Against Family Violence. https://catalogue.nla.gov.au/catalog/5009611.

[bibr32-10778012231214775] InTouch Multicultural Centre Against Family Violence. (2018). *Submission to the Royal Commission into Family Violence*. https://www.rcfv.com.au/getattachment/50C16832-F42A-481D-A042-AC388D73B2BE/inTouch-Multicultural-Centre-Against-Family-Violence-.

[bibr33-10778012231214775] InTouch Multicultural Centre Against Family Violence. (2021). *Criminalisation of coercive control: Should coercive control be a criminal offence in Victoria? Position Paper*. https://intouch.org.au/wp-content/uploads/2022/11/inTouch-Position-Paper-Criminalisation-of-Coercive-Control-2021-FINAL.pdf.

[bibr34-10778012231214775] Jones B. (2020). The politics of care: A case study of domestic violence in Aceh. In McGregor K. Dragojlovic A. Loney H. (Eds.), Gender, violence and power: Indonesia across time and space. Routledge.

[bibr35-10778012231214775] Jones B. Inayatillah I. Nursiti N. (2022). Using Aceh’s Qanun to expand protection for domestic violence victims. Australian Journal of Asian Law, 23(2), 63–72. https://ssrn.com/abstract=4284337 .

[bibr36-10778012231214775] Kalapac V. (2016). inLanguage, inCulture, inTouch: Integrated model of support for CaLD women experiencing family violence. Jean Hailes for Women’s Health. https://lsbc.vic.gov.au/sites/default/files/2020-09/inLanguage,%20inCulture,%20inTouch%20Report,%20InTouch%20Multicultural%20Centre%20Against%20Family%20Violence,%20December%202016.pdf .

[bibr37-10778012231214775] Kim M. (2018). From carceral feminism to transformative justice: Women-of-colour feminism and alternatives to incarceration. Journal of Ethnic & Cultural Diversity in Social Work, 27(3), 219–233. 10.1080/15313204.2018.1474827.

[bibr38-10778012231214775] Lean T. Rule L. Porter A. Whitaker A. (2021). John Barry Memorial Lecture: Abolition on Indigenous land [Video]. *YouTube*. https://www.youtube.com/watch?v=peA6_WdIbtE.

[bibr39-10778012231214775] Machado A. Santos A. Graham-Kevan N. Matos M. (2017). Exploring help seeking experiences of male victims of female perpetrators of IPV. Journal of Family Violence, 32, 513–523. 10.1007/s10896-016-9853-8.

[bibr40-10778012231214775] Mantle G. Hermans M. (2020). Criminalising coercive control is not the answer: An abolitionist critique. Honi Soit. https://honisoit.com/2020/10/criminalising-coercive-control-is-not-the-answer-an-abolitionist-critique/ .

[bibr41-10778012231214775] Masson A. (2020). A critique of anti-carceral feminism. Journal of Women’s Studies, 21(3), 64–76. https://vc.bridgew.edu/jiws/vol21/iss3/6 .

[bibr42-10778012231214775] Monash Gender and Family Violence Prevention Centre. (2020). *Criminalisation of coercive control: Research brief*. https://bridges.monash.edu/articles/online_resource/Criminalisation_of_Coercive_Control_-_Research_Brief/13017743.

[bibr43-10778012231214775] Mugford J. (1989). *Domestic violence*. Australian Institute of Criminology. https://www.aic.gov.au/publications/vt/vt2.

[bibr44-10778012231214775] Multicultural Centre for Women’s Health. (2015). *Submission to the Royal Commission into Family Violence (Victoria)*. https://www.mcwh.com.au/wp-content/uploads/MCWH-Submission-RCFV-May-2015.pdf.

[bibr45-10778012231214775] Muslim Women Australia. (2021). *Submission to the NSW Joint Select Committee on Coercive Control*. https://mwa.org.au/submissions-and-reports/submission-to-the-joint-select-committee-on-coercive-control/.

[bibr46-10778012231214775] Nancarrow H. Thomas K. Ringland V. Modini T. (2020). Accurately identifying the “person most in need of protection” in domestic and family violence law: Report. *ANROWS*. https://anrowsdev.wpenginepowered.com/wp-content/uploads/2019/10/Nancarrow-PMINOP-RR.3.pdf.

[bibr47-10778012231214775] National Domestic and Family Violence Bench Book. (2022). *People from culturally and linguistically diverse backgrounds*. https://dfvbenchbook.aija.org.au/vulnerable-groups/people-from-culturally-and-linguistically-diverse-backgrounds/.

[bibr48-10778012231214775] No To Violence. (2019). *Predominant aggressor identification and victim misidentification* . https://ntv.org.au/wpcontent/uploads/2020/06/20191121-NTV-Discussion-Paper-Predominant-Aggressor-FINAL.pdf.

[bibr49-10778012231214775] NSW Department of Communities and Justice. (2022). *NSW passes landmark coercive control reform*. https://www.dcj.nsw.gov.au/news-and-media/media-releases/2022/nsw-passes-landmark-coercive-control-reform.html.

[bibr50-10778012231214775] One Woman Project. (2021). *Why criminalising coercive control in Australia poses danger for First Nations women*. https://www.onewomanproject.org/first-nations/why-criminalising-coercion-and-control-in-australia-poses-danger-for-first-nations-women.

[bibr51-10778012231214775] Otter C. Bosanko M. Hocking A. (2021). * What is coercive control? Analysis & Policy Observatory *. https://apo.org.au/node/317274.

[bibr52-10778012231214775] Parliament of New South Wales. (2020). *Joint select committee on coercive control*. https://www.parliament.nsw.gov.au/committees/listofcommittees/Pages/committee-details.aspx?pk=271.

[bibr53-10778012231214775] Pittaway E. Muli C. Shteir S. (2011). I have a voice—Hear me!” Findings of an Australian study examining the resettlement and integration experience of refugees and migrants from the Horn of Africa in Australia. Refuge: Canada’s Journal on Refugees, 26(2), 133–146. 10.25071/1920-7336.32084.

[bibr54-10778012231214775] Queensland Women’s Safety and Justice Taskforce. (2021). Hear her voice – Report one - Addressing coercive control and domestic and family violence in Queensland. https://www.womenstaskforce.qld.gov.au/publications.

[bibr55-10778012231214775] Quilter J. (2020). Evaluating criminalisation as a strategy in relation to non-physical family violence. In McMahon M. McGorrery P. (Eds.), Criminalising coercive control: Family violence and the criminal law (pp. 111–134). Springer.

[bibr56-10778012231214775] Reeves E. (2021). 'I’m not at all protected and I think other women should know that, that they’re not protected either’: Victim–survivors’ experiences of ‘misidentification’ in Victoria’s family violence system. International Journal for Crime, Justice and Social Democracy, 10(4), 39–51. 10.5204/ijcjsd.1992.

[bibr57-10778012231214775] Reeves E. McGowan J. Scott B. (2023). ‘It was dangerous, corrosive and cruel but not illegal’: Legal help-seeking behaviours amongst LGBTQA+ domestic and family violence victim-survivors experiencing coercive control in Australia. Journal of Family Violence. 10.1007/s10896-023-00569-9.

[bibr58-10778012231214775] Robinson C. (2022, 25 October). Why Aboriginal women fear NSW’s new coercive control laws could do more harm than good. *The Guardian*. https://www.theguardian.com/society/2022/oct/25/why-aboriginal-women-fear-nsws-new-coercive-control-laws-could-do-more-harm-than-good.

[bibr59-10778012231214775] Sentencing Advisory Council. (2022). Sentencing breaches of family violence intervention orders and safety notices: Third monitoring report. Victoria State Government. https://www.sentencingcouncil.vic.gov.au/publications/sentencing-breaches-of-family-violence-intervention-orders-and-safety-notices .

[bibr60-10778012231214775] Shuard J. (2021). Monitoring Victoria’s family violence reforms: Accurate identification of the predominant aggressor. Office of the Family Violence Reform Implementation Monitor.

[bibr61-10778012231214775] Smith D. (2008). Intersectionality as buzzword: A sociology of science perspective on what makes a feminist theory successful. Feminist Theory, 9(1), 67–85. 10.1177/1464700108086364.

[bibr62-10778012231214775] Taylor J. C. Bates E. A. Colosi A. Creer A. J. (2022). Barriers to men’s help seeking for intimate partner violence. Journal of Interpersonal Violence, 37(19-20), 18417–18444. 10.1177/08862605211035870. PMC9554285 34431376

[bibr63-10778012231214775] Tsui V. Cheung M. Leung P. (2010). Help-seeking among male victims of partner abuse: Men’s hard times. Journal of Community Psychology, 38(6), 769–780. 10.1002/jcop.20394.

[bibr64-10778012231214775] University of Queensland Pro Bono Centre. (2020). Domestic and Family Violence in Culturally and Linguistically Diverse (CALD) Communities.

[bibr70-10778012231214775] Victoria Police. (2022). *Family violence intervention orders*. https://www.police.vic.gov.au/intervention-orders.

[bibr65-10778012231214775] Victorian Aboriginal Legal Service. (2022). *Addressing coercive control without criminalisation: Avoiding blunt tools that fail victim-survivors*. https://www.vals.org.au/wp-content/uploads/2022/01/Addressing-Coercive-Control-Without-Criminalisation-Avoiding-Blunt-Tools-that-Fail-Victim-Survivors.pdf.

[bibr66-10778012231214775] Victorian Foundation for Survivors of Torture. (2015). *Submission to the Royal Commission into Family Violence*. https://www.foundationhouse.org.au/wp-content/uploads/2015/06/VFST_RCFV_Sub_June_2015.pdf. 10.3109/00048679509064955 8573050

[bibr67-10778012231214775] Victorian Government. (2022). *Presentations of family violence in different relationships and communities*. https://www.vic.gov.au/maram-practice-guides-foundation-knowledge-guide/presentations-family-violence-different.

[bibr68-10778012231214775] Victorian Multicultural Commission. (2015). *Submission to the Victorian Royal Commission into Family Violence*. https://www.multiculturalcommission.vic.gov.au/submissions-to-government#royal-commission-into-family-violence.

[bibr69-10778012231214775] Victorian Royal Commission into Family Violence. (2016). *Summary and recommendations*. http://rcfv.archive.royalcommission.vic.gov.au/MediaLibraries/RCFamilyViolence/Reports/Final/RCFV-Summary.pdf.

[bibr71-10778012231214775] Walklate S. Fitz-Gibbon K. (2021). Why criminalise coercive control? The complicity of the criminal law in punishing women through furthering the power of the state. International Journal for Crime, Justice and Social Democracy, 9(4), 1–12. 10.5204/ijcjsd.1829.

[bibr72-10778012231214775] Walklate S. Fitz-Gibbon K. McCulloch J. (2018). Is more law the answer? Seeking justice for victims of intimate partner violence through the reform of legal categories. Criminology & Criminal Justice, 18(1), 115–131. 10.1177/1748895817728561.

[bibr73-10778012231214775] Wangmann J. (2022). Law reform processes and criminalising coercive control. Australian Feminist Law Journal, 48(1), 57–86. 10.1080/13200968.2022.2138186.

[bibr74-10778012231214775] Watego C. Macoun A. Singh D. Strakosch E. (2021). Carceral feminism and coercive control: When Indigenous women aren’t seen as ideal victims, witnesses or women. *The Conversation*. https://theconversation.com/carceral-feminism-and-coercive-control-when-indigenous-women-arent-seen-as-ideal-victims-witnesses-or-women-161091.

[bibr75-10778012231214775] Western Australia Commissioner for Victims of Crime. (2023). Coercive control consultation. www.wa.gov.au/organisation/department-of-justice/commissioner-victims-of-crime/coercive-control-consultation?msclkid=20bee396d0ff11ec8797b0d96e3b3809.

[bibr76-10778012231214775] Women’s Legal Services Victoria. (2020). Policy brief: Justice system response to coercive control. https://www.womenslegal.org.au/∼womensle/wp-content/uploads/2021/04/CoerciveControl_policy_brief.pdf.

[bibr77-10778012231214775] Wright S. E. (2023). Navigating the disjuncture between domestic and family violence systems: Australian Muslim women’s challenges when disclosing violence. Australian Feminist Law Journal, 48(2), 321–347. 10.1080/13200968.2023.2170893.

[bibr78-10778012231214775] YourSAy. (2021). Criminalising coercive and controlling behaviours. https://yoursay.sa.gov.au/control.

